# Team behaviors in emergency care: a qualitative study using behavior analysis of what makes team work

**DOI:** 10.1186/1757-7241-19-70

**Published:** 2011-11-15

**Authors:** Pamela Mazzocato, Helena Hvitfeldt Forsberg, Ulrica von Thiele Schwarz

**Affiliations:** 1 Medical Management Centre, Department of Learning, Informatics, Management, and Ethics, Karolinska Institutet, Stockholm, Sweden; 2 Department of Psychology, Stockholm University, Stockholm, Sweden

**Keywords:** Accident and emergency department, communication, emergency department, implementation, process redesign, Sweden, teamwork

## Abstract

**Objective:**

Teamwork has been suggested as a promising approach to improving care processes in emergency departments (ED). However, for teamwork to yield expected results, implementation must involve behavior changes. The aim of this study is to use behavior analysis to qualitatively examine how teamwork plays out in practice and to understand eventual discrepancies between planned and actual behaviors.

**Methods:**

The study was set in a Swedish university hospital ED during the initial phase of implementation of teamwork. The intervention focused on changing the environment and redesigning the work process to enable teamwork. Each team was responsible for entire care episodes, i.e. from patient arrival to discharge from the ED. Data was collected through 3 days of observations structured around an observation scheme. Behavior analysis was used to pinpoint key teamwork behaviors for consistent implementation of teamwork and to analyze the contingencies that decreased or increased the likelihood of these behaviors.

**Results:**

We found a great discrepancy between the planned and the observed teamwork processes. 60% of the 44 team patients observed were handled solely by the appointed team members. Only 36% of the observed patient care processes started according to the description in the planned teamwork process, that is, with taking patient history together. Beside this behavior, meeting in a defined team room and communicating with team members were shown to be essential for the consistent implementation of teamwork. Factors that decreased the likelihood of these key behaviors included waiting for other team members or having trouble locating each other. Getting work done without delay and having an overview of the patient care process increased team behaviors. Moreover, explicit instructions on when team members should interact and communicate increased adherence to the planned process.

**Conclusions:**

This study illustrates how behavior analysis can be used to understand discrepancies between planned and observed behaviors. By examining the contextual conditions that may influence behaviors, improvements in implementation strategies can be suggested. Thereby, the adherence to a planned intervention can be improved, and/or revisions of the intervention be suggested.

## Introduction

### Background

Overcrowding and excessively long waits are a concern for emergency departments (ED) around the world [1-3]. Factors contributing to these problems include lack of resources and specialist competence, delays in diagnostic processes and lack of inpatient beds, but also inefficient working procedures [2,4]. It has been argued that simply adding resources (such as hospital beds) is not sufficient to fix flow problems [5]. One of the interventions designed to help improve patient and work flow in the ED is multidisciplinary teamwork [5,6]. Previous research has suggested that redesigning care processes using a teamwork approach is related to time savings and improved patient flow [7-9]. Accordingly, in this study, team was defined as a group of three care-givers forming a team that lasted for the whole work-shift. The team was responsible for entire care episodes, i.e. from patient arrival to patient discharge from the ED. This differs from other teams described in the literature, e.g. trauma/emergency teams that handle acute patients, or triage or rapid assessment teams [10-13]. Also, it differs from interventions focusing on communication- and/or team *training *[14,15] as this study focuses on how patient- and work flow can be organized and managed rather than on individual skill training.

### Importance

Regardless of the content of teamwork interventions, these need to be translated into practice in order to achieve target outcomes [16,17]. Whether interventions succeed or not do not only depend on the content of the intervention but also to what degree the intervention is implemented [18]. Implementation requires behavior change, which is one of the most challenging issues in implementation research. There has been a lack of theoretical models within implementation science for understanding how behavior change can be achieved [19,20]. Operant psychology and the use of behavior (or functional) analysis is a theory of human behavior. Given the assumption that implementation requires behavior change, we propose that behavior analysis can be a theoretically driven way of examining teamwork and identifying facilitators for, and hinders to, implementation of teamwork.

### Goals

This study examines teamwork process (the behaviors team members perform to handle each care episode) observed during the introduction of teamwork. The aim is to use behavior analysis to qualitatively explore the differences between planned teamwork process and actual team behaviors, and to analyze any discrepancies between them.

We set out to answer the following research questions:

1. How did teamwork play out in practice compared to the planned teamwork process?

2. Which key team behaviors were most important in implementing the new teamwork process?

3. Which contingencies increased or decreased the likelihood of these key behaviors?

## Method

### Theoretical model

According to operant psychology, which forms the theoretical basis for behavior analysis, behavior is determined by its antecedents (or discriminative stimuli) and consequences [21]. Antecedents come before the behavior and act as a signal, or trigger, of the behavior. As such, the antecedents are necessary for the behavior to take place. However, this is not sufficient: what happens after the behavior, as a result of it, determines whether it is likely to be repeated in a similar situation in the future. The likelihood of a behavior increases if it is followed by something pleasant or rewarding (positive reinforcement) or if a negative condition is stopped or avoided (negative reinforcement). The likelihood of a behavior decreases when it is followed by a negative condition or experience (punishment) or if it has no consequence (extinction). In behavior analysis, antecedents and consequences, e.g. the contingencies for key behaviors, are investigated. The focus is on the function of the behavior, e.g. what happens as a consequence of the behavior and how that, in turn, affects the likelihood for behavior in the future. Using behavior analysis, people's motivation to engage or not engage in a behavior can be understood. From this, hypotheses about which contingencies that will maximize the likelihood of key behaviors can be put forth, and tested in an empirical, iterative process.

### Study design

This study is part of a mixed-methods research project investigating the effects of Teamwork on Efficiency, Patient safety, Patient satisfaction and Personnel work environment (the TEPPP study) in an ED. A qualitative observation study was designed to fit the exploratory nature of this sub-study. It was conducted in June 2010 as an ED implemented teamwork. The project was approved by the Regional Ethical Review Board.

### Setting

The study setting is an ED in Sweden. Approximately 50,000 patients visit the ED each year (192 per 100,000 inhabitants), and it is divided into four different specialties: internal medicine, trauma, orthopedic, and surgical care. The ED employs approximately 120 nurses and 20 physicians (residents) (registered nurses (RN) and nurse assistants), who rotate between the sections, while approximately 200 of the physicians at the hospital work shifts in the ED. All team physicians in this study had previous experience in emergency medicine and from working in this specific ED.

Traditionally, the work process for each care episode started with a spot-check nurse at the registration desk making an initial and preliminary assessment of urgency and specialty. Another RN then decided on which patient to be handled next, and the nurse assistant walked the patient into a patient room and started taking vital signs. The RN then began taking patient history and performed a triage. Then, the first available physician examined the patient. In the proceeding work process, one nurse was responsible for documentation in Electronic Medical Record (EMR) while another handled ordinations and performed the nursing interventions in collaboration with a nurse assistant. This means that each physician worked with any nurse and nurse assistant available, and the patient met two nurses before seeing the physician, resulting in duplication of work tasks, waste in terms of time spent looking for each other and multiple handovers.

### Intervention - Planned team process and behaviors

Teamwork was implemented in the specialty of internal medicine of the ED, Monday through Friday, 8 am to 9 pm. Each day, four teams were scheduled: one between 8 am and 4 pm, one between 10 am and 7 pm, and two between 1 pm and 9 pm. Each team consisted of a physician (with varying experience), a registered nurse and a nurse assistant.

Following the model outlined by Braksick [22], implementers (business consultants and work groups consisting of ED employees and management) brainstormed team behaviors and pinpointed those believed to be most important in the teamwork process. These were then pilot tested and further developed. Thus, the behaviors in the planned teamwork process were outlined by implementers rather than the researchers and they were based on a generic model from the performance management literature, rather than on the current literature on teamwork behaviors.

**Figure 1:**
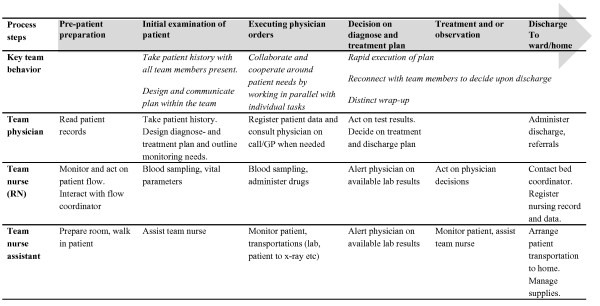
**The planned process for teamwork with the behaviors specified for each member of the team**.

The final version of the team behaviors can be found in Figure 1, along with the overall teamwork process and the behaviors of each team member specified.

Rather than focusing on specific skill training of individuals, this project focused on process redesign, e.g. changing the environment and work routines to better enable teamwork. The overall idea was to have the same team handling the whole care episode. A number of strategies were used to facilitate the implementation process. 1) Each employee participated in a half day workshop focusing on interactive discussions on necessary adjustments, personal and organizational, for making teamwork possible. 2) The teamwork process and the different roles were outlined in a handbook distributed to each employee at the ED along with 3) a flashcard summarizing the most important behaviors. 4) Nurse supervisors provided feedback and support on teamwork, during and after work shifts. 5) The new role of a flow manager was developed with the task to assign patients to teams and to monitor and coordinate patient flow. The specialist was given similar responsibilities.

### Data collection and processing

*Observations: *In June 2010, observations were performed on three consecutive days the second week after the implementation of teamwork, between 8 am and 4:30 pm. The days and time were chosen by the observers, independent of the ED. The observations totaled 50 hours. The early phase of implementation was chosen in order to make the results useful for the implementers in the implementation process and to provide a description of the independent variable in the effectiveness studies in the TEPPP-project. Each day, two of the three observers (UvTS, HHF, PM) were present. During the mornings, one observer "shadowed" a team, following one or several of the team members during their work. The other observer was stationed at the central desk, situated at the center of the ED, following the same team from there and making general observations of patients and workflow as well as other activities concerning the team. During lunches and breaks, the observers focused on how this affected teamwork. During the afternoon, when there were several teams (generally four), each observer shadowed a team. The observers alternated roles. The observers also posed questions to the team members and other coworkers at the ED, in order to clarify issues that were not clear from observations alone, e.g. "What are you doing right now?", "Do you know where the other members of your team are?" and "Do you have a plan for this patient? The observations were structured around the planned team behaviors (see Figure 1) and documented in an observation scheme. The final structure of the scheme was reached after a pilot test during an observation prior to the actual data collection phase.

The observation notes were transcribed by each observer. When several observers had observations relating to the same care episode, these were combined. The EMR was used to validate the quantitative data from the observations, e.g. number of patients, lead times, etc.

## Results

### Characteristics of patients and teams observed

During the fifty hours of observations at the ED medical section, we followed the 44 patients (18 women and 25 men, 1 missing data on gender) that the team's cared for. Common reasons for seeking care at the ED were chest pain, respiratory-related problems, low general state of health and dizziness. The team physician was an intern, resident or specialist. Each day, two to four teams were observed. Team members differed between days. The attending specialist was the same person through all days of observations.

### Observed teamwork process in numbers

Of the 44 observed patients, a total of 60 percent were handled solely by team members throughout the care episode. However, patient history was taken with all team members present (one of the key behaviors) for only 36 percent of the patients. Overall, there was great variation between the three days of observations in terms of patient load and adherence to the planned teamwork process.

### Planned versus actual team behaviors

Below is a description of how teamwork was actually performed, in terms of deviation from the planned teamwork process.

#### Take patient history with the whole team present

Although this team behavior was only performed in a minority of the care episodes, it was still the most successfully implemented part. Deviations from the planned teamwork process involved the physician taking the patient history alone or the nurse starting to take the history as part of a nurse triage. Deviations also appeared as teams took over the care for ambulance arrivals, whose patient history had already been taken by the trauma team.

#### Design a plan and communicate it within the team

Although the physicians often communicated a plan to the patient, sometimes with the other team members present, they seldom discussed the plan with the team without the patient present. Also, the physicians mainly focused on immediate actions such as the next step, e.g. blood samples to be collected, X-rays to be taken, etc. This lack of specificity led the nurses to ask about, or suggest, suitable actions to take.

#### Collaborate and cooperate around patient needs

When the caregivers met as a team, the collaboration was generally satisfactory. They communicated about patient needs, asked clarifying questions concerning treatment plans and made the team stay on track by asking questions and probing, e.g. "We have the lab-results; what's next?" However, after the team members had left the examination room and were "on their own", they tended to get stuck with tasks not related to the team's patients. The teams ended up trying to localize each other, and if they could not find the other team members they proceeded on their own, with either the team's patients or other tasks. In sum, the team members worked in parallel but not only with tasks related to their patients, and had trouble reconnecting as a team.

#### Rapid execution of plan

Discussion and decision on discharge plans were seldom initiated by the physician. Instead, it was often the nurses who asked about the discharge plan and status. At other times, the physicians finalized the discharge plan without communicating it to other team members. Administrative work around discharge was delayed, often due to poor communication or to team members' occupation with other tasks and/or patients.

### Key team behaviors and contingencies affecting implementation of teamwork

Based on the observations of the cases when the teams managed to maintain teamwork throughout the care episode, we inductively identified commonalities in team behaviors. The revised key behaviors are outlined in Table 1 along with the behavior analysis of the most important conditions that triggered the behaviors (antecedents) and factors that increased or decreased the likelihood of the behavior (consequence).

**Table 1:** Contingencies affecting team efficiency.

Antecedents	Key Behaviors	Consequences
Team members communicate about decision to start the next care episode	**Take patient history together**	Team physician saves time by not having to repeat patient information and physician orders (+)
Team members free from work tasks related to previous patients		Team nurses know what to do (e.g. which physician orders to act on) (+)
All team members present		Team members can solve problems immediately (+)
Nurses inform patients that patient history will be taken with all team members present		Team members perceive that they are being watched and potentially evaluated by other team members (-)
		Team members have to wait for each other (-)
The assigned team room available	**Go to assigned team room**	Team members find each other and can perform task without unnecessary delay (+)
Team members have or need to communicate and coordinate		Team members lack overview over total patient flow in the ED (-)
		Team members find no available work station in the assigned team room (-)
		Team members perceive that they are being watched and potentially evaluated by other team members (-)
Team members have or need new information on an ongoing patient	**Verbal communicate with team members**	Team members know what tasks have been performed and what to do next (+)
Team members need to revise the plan for an ongoing patient		Team members have an overview over the team patients throughout the process (+)
Physician decides on discharge of patient		Team members perform tasks without delays and the results of one's work are visible (+)
Team members need to revise the patient flow or attend to tasks unrelated to team tasks		Team members perceive that they are being watched and potentially evaluated by other team members (-)
Team members are assigned other tasks unrelated to team members		Team members perceive their own tasks and work pace to be influenced by others (-)
Team members have access to each other		

Pinpointed behaviors that are essential for successful teamwork and conditions triggering the behaviors (antecedents) and increasing (+) or decreasing (-) the likelihood for the behaviors. unless otherwise specified.

The first revised key behavior, taking patient history as a team, corresponded to the first key behavior described by the implementers. The second, communicating with team members, and the third, meeting in an assigned team room (using the team room as a work station), were part of the planned teamwork process but not highlighted as key behaviors as such. However, when these behaviors were not performed this was related to a disruption of teamwork. In fact, when the teams failed to take patient history together, teamwork was not performed at all.

Since the team members were dependent on each other for the completion of work tasks, one key to enabling teamwork was their easy access to each other. This highlights the need to have an available space where the team can meet, as it allows team members to find each other quickly. As seen in Table 1, once the team members met, work tasks were completed and an overview of the whole care episode was achieved, thus reinforcing teamwork. On the other hand, inter-relational aspects such as being observed and potentially evaluated by others, as well as having less autonomy over the work tasks and pace decreased the likelihood of working in a team. Table 1 also shows that for communication to be effective, it had to be initiated in response to crucial steps in the care process, that is, having clear antecedents.

## Discussion

This study shows that when teamwork was implemented in the clinical setting there was a discrepancy between the planned teamwork process and the actual teamwork behaviors. This was not unexpected, given that the study was done during the early phases of implementation. However, this illustrates the importance of closely monitoring actual behaviors as implementation takes place. By using behavior analysis, this study goes beyond merely describing the extent of such discrepancy by illuminating plausible key behaviors, and the contingences that made these behaviors more likely. This information can be used either to adjust the planned process in accordance with what is practically possible to perform, or to increase the fidelity to the planned process by managing the contingences in order to make the target behaviors more likely to be performed. In the end, these steps are necessary to take before it is reasonable to evaluate the effectiveness of an intervention.

In this study, the revised key behaviors were taking patient history together, meeting in a defined team room, and communicating with team members. When these were performed, the members were able to continuously work together throughout the care episode. Although these behaviors should not be interpreted as generic team behaviors, they may provide an example of how efficient team work can be understood and how the behaviors may be influenced by immediate consequences. The likelihood of the key behaviors was affected by social, personal, and environmental factors. In particular, to increase the likelihood of these three team behaviors, it was important to avoid negative consequences such as having to wait for other team members, having trouble locating each other, and being watched and potentially judged by others. It was also important to be explicit about when team members should interact and communicate.

Taking patient history together has previously been identified as an important part of teamwork [23]. This may allow the team to get a shared situational awareness, defined as an individual's awareness of important care-related information and events [24]. This awareness is particularly important in work situations with high cognitive demands [25], and has previously been related to high performance in trauma teams [26]. In contrast to when staff works independently in the ED, taking patient history together reduces the need to hand over patient information [27] as well as the work duplication caused by patients giving their history numerous times to different staff members [28]. However, although taking patient history together may be beneficial from a patient safety perspective and may be more effective in some aspect, it may also be time consuming if one or several of the team members is not able to be engaged in any appropriate task during the activity. More research is needed on the circumstances that make taking patient history contribute to efficient teamwork.

The second key team behavior, meeting in a team room, allowed team members to find each other easily. Besides saving time, this also helped them coordinate their work in a timely manner and reminded them to communicate with each other. Moreover, the team room provided an area where the team members could communicate without patients nearby, which has been suggested as a way to reduce misunderstanding and mistakes [28]. Given that the ED has previously been described as cognitively challenging [29], the team room may also lessen these cognitive demands.

Given the importance of inter-professional communication within the ED [30] and given how frequent staff in the ED is involved in communication [31], the third key behavior, communicating with team members, is not surprising. Effective communication has notoriously been difficult to implement [32]. In the planned teamwork process in this study, the antecedents for communication were not specified. From this follows that although team members did communicate, this communication was frequently delayed and was not always sufficient for effective teamwork. This indicates that besides focusing on communication behaviors as such, it may be equally important to target when communication within the team is essential, e.g. clarify the antecedents for team communication. The most important antecedents for communication we found are similar to those highlighted as particularly important by Risser [33]. Besides providing a cue for the initiation of communication, these can all be seen as means to accomplish a common situational awareness.

In summary, the key team behaviors found in this study were similar to those described in previous studies on communication and team training [15,18,34,35]. However, these studies focus on training, whereas the intervention described here focuses on environmental change. Thus, the fundamental assumption behind the intervention we studied, as well as our methodological approach (i.e. behavior analysis), is that individual behaviors are shaped by the environment in which they appear. Thus, it is assumed that team members have sufficient interpersonal skills to engage in productive team behavior. The importance of adjusting environmental factors, including physical layout and managerial support, to facilitate the implementation of teamwork has been stressed previously [14,18]. Also, this study focuses on the immediate consequences, which according to theory are most likely to affect behavior. Thereby, it provides an additional perspective to previous studies looking at more general principles for successful implementation of teamwork such as institution-level incentives to train and multi-professional training of staff in their units [36].

### Limitations

A qualitative study has drawbacks in terms of conclusions on relationships in comparison to a quantitative approach. However, given that the aim of the study was to explore the implementation of teamwork, a qualitative method using direct observation was deemed suitable. It is more objective than self-ratings and has been described as particularly useful to investigate complex interactions between individuals, teams and organizational precursors of teamwork performance [17]. Structured observation scheme, immediate note-taking, parallel observations and clarification questions during observations were used to minimize observer effects such as selective memory effects and observer misconceptions. However, this also made the observers more visible, which may have contributed to the observer-expectancy effect. Although not completely avoidable, this effect was limited by getting the employees accustomed to the observers' presence through pilot observations and by emphasizing that the researchers' interest was not in how well the team members performed but rather on how it was possible to translate the planned process into practice. In this sense, it was not so much the team members who were scrutinized but the implementers.

Observations were made on different weekdays, at different times of the day, on different teams and on team doctors with different levels of experience. Moreover, the observation days differed in terms of patient inflow, workload and number of hospital beds available. Although such factors have been shown to affect the efficiency of teamwork in other studies [18], it was beyond the scope of this study to investigate the relative influence of such factors. Rather, the goal was to tap into a variety of situations rather than minimize variance.

The analysis of qualitative data may be subject to interpretation bias. However, the use of an analytic model based on a well-established theoretical framework minimizes this risk. Also, the results were validated by key informants, including teamwork implementers and behavior analysis professionals. The results of this study were also fed back to the ED to help them improve future teamwork processes. It also contributed to continuous learning in the ED.

Although the ED in this study is typical in terms of size and work tasks, and the challenges its employees face are similar to those of other EDs in the industrialized world, the single case study design entails limitations in terms of external validity. Hence, this study can tell what might be possible, but not necessarily what is likely, in other settings. However, the results of this study can serve as hypotheses to be tested in other settings using a multiple case study design and could be particularly useful where the implementation of teamwork has been difficult.

## Conclusions

This study shows how analysis of contingencies (the antecedents and consequences) for key team behaviors can contribute to the understanding of why some aspects of a teamwork process are implemented with high fidelity and others are not. This understanding can be used to suggest improvements in implementation strategies, and thereby improving the adherence to the planned intervention, and/or for revisions of the intervention. The fact that the focus in behavior analysis is on the function of observable behaviors makes it particularly useful in implementation, in that it readily translates into changes in practice.

## Competing interests

The authors declare that they have no competing interests.

## Authors' contributions

PM, HHF, and UvTS were involved in the original conception and design of the article. UvTS obtained research funding. PM, HHF, and UvTS have contributed equally to the collection, analysis, and interpretation of the data. PM, HHF, and UvTS have also contributed equally to drafting and revising the manuscript. All authors have approved the final version of the paper and take responsibility for the paper as a whole.
